# *Trichoderma asperellum* Ta1 Alleviates Root Rot Caused by *Fusarium solani* and Promotes the Growth of *Panax notoginseng*

**DOI:** 10.3390/jof11120879

**Published:** 2025-12-11

**Authors:** Yue Gu, Jie Deng, Youyu Li, Diqiu Liu

**Affiliations:** Faculty of Life Science and Technology, Kunming University of Science and Technology, Kunming 650500, China; gy18085780411@126.com (Y.G.); dj2826122825@126.com (J.D.); 18589372640@163.com (Y.L.)

**Keywords:** *Panax notoginseng*, *Trichoderma asperellum*, root rot, biological control, multi-omics analysis

## Abstract

*Panax notoginseng* is a traditional medicinal and food-homologous plant in China; however, root rot, primarily caused by *Fusarium solani*, has become a devastating disease that severely compromises the yield and quality of *P. notoginseng*. In this study, a strain of *Trichoderma asperellum* (designated as Ta1) was isolated from the rhizosphere soil of healthy *P. notoginseng*. In vitro, Ta1 exhibited strong antagonistic activity against *F. solani*, with a growth inhibition rate of 65.53%, and the spore germination of *F. solani* was inhibited. In pot experiments, Ta1 reduced the incidence of root rot, and the biocontrol efficiency was around 59.55%. Additionally, Ta1 improved the growth vigor of *P. notoginseng* plants, resulting in significant increases in key agronomic traits, photosynthetic parameters, levels of primary metabolites, concentrations of plant growth-promoting hormones, and the accumulation of bioactive saponins. Transcriptomic and metabolomic analyses revealed that Ta1 upregulates key genes involved in the biosynthesis of phenylpropanoids, monoterpenoids, and diterpenoids, as well as in jasmonic acid signaling. Given its dual functions in disease control and growth promotion, Ta1 represents a promising biological control agent with great potential for application in *P. notoginseng* cultivation, contributing to the enhancement of its yield and quality.

## 1. Introduction

*Panax notoginseng* (Burk.) F.H. Chen is a traditional medicinal and food-homologous plant in China, highly valued for its saponins [1]. Although saponins possess certain toxicity and can cause adverse drug reactions, including fever, local bleeding, and allergic purpura, they are widely utilized in clinical practice for the treatment of cardiovascular diseases due to their pharmacological activities [2]. For example, the active ingredients of Xuesaitong soft capsules are saponins derived from *P. notoginseng*. However, root rot, primarily caused by *Fusarium solani* (*Neocosmospora solani*), poses a significant threat to the yield and quality of *P. notoginseng* [3]. This condition leads to necrosis in the roots of *P. notoginseng*, severely impairing saponin biosynthesis and diminishing its medicinal and edible value [4]. In agricultural practices, chemical pesticides are frequently employed to combat root rot; however, their widespread use raises serious environmental and health concerns, contributing to soil pollution and posing risks to human well-being [5].

Biological control strategies, which utilize antagonistic microorganisms to suppress plant pathogens, present an environmentally sustainable alternative for managing root rot in crops [6]. For instance, antagonistic microorganisms such as *Trichoderma harzianum* and *Bacillus amyloliquefaciens* have been effectively employed to mitigate *Fusarium oxysporum*-induced root rot in *Atractylodes chinensis* through mechanisms including competitive exclusion, nutrient competition, and preemptive colonization of infection sites. Similarly, *Hannaella sinensis* has demonstrated significant inhibitory effects on the spore germination and germ tube elongation of *Penicillium expansum* [7]. Furthermore, the extract from *Arcopilus aureus* SQGX-6 has been shown to enhance the resistance of *Lycopersicon esculentum* to *Botrytis cinerea* by improving the plant’s capacity to scavenge reactive radicals and increasing the activity of peroxidase (POD) and phenylalanine ammonia lyase (PAL) [8]. In *P. notoginseng*, *Bacillus velezensis* B19 has demonstrated the ability to inhibit the growth of *F. solani* and *F. oxysporum*, thereby reducing the incidence of root rot in *P. notoginseng* [9]. *Bacillus subtilis* Pn1 enhanced the phenylpropanoid and flavonoid metabolism of *P. notoginseng*, increasing its resistance to *F. solani*, and also promoted the growth of *P. notoginseng* plants [10].

*Trichoderma* spp. are saprophytic fungi widely used worldwide as biocontrol agents and growth promoters [11]. Currently, over 370 *Trichoderma* spp., including *T. harzianum*, *Trichoderma koningiopsis*, *Trichoderma viride*, and *Trichoderma asperellum*, are utilized in agricultural practices for disease control [12,13]. Many *Trichoderma* spp. assist plants in defending against pathogenic fungi through various mechanisms, including parasitism, nutrient competition, and the production of volatile metabolites [14,15,16]. For instance, the volatile organic compounds from *Trichoderma atroviride* ATR697 have been shown to inhibit the growth of *F. oxysporum*, and its spore suspension effectively reduced the incidence of anthracnose in pepper [17]. In dual-plate assays with *Fusarium pseudograminearum* (Fg), *Trichoderma longibrachiatum* (TG1) exhibited significant upregulation of cell wall-degrading enzymes, such as β-1,6-glucan synthase, endochitinase, and chitinase, leading to degradation of the Fg cell wall and subsequent growth inhibition over 14 days [18]. In *P. notoginseng*, *Trichoderma gamsii* YIM PH30019 controls root rot by mycoparasitizing *Phoma herbarum* and *Fusarium flocciferum*, as well as producing volatile organic compounds [19]. Additionally, *T. atroviride* 1126, *T. harzianum*, and *T. koningiopsis* T5-1 have been shown to suppress root rot in *P. notoginseng* by inhibiting the growth of *Phytophthora cactorum* [20].

Despite the demonstrated efficacy of various biocontrol agents against *P. notoginseng* root rot, the intricate mechanisms governing the interactions among antagonistic microorganisms, pathogens, and *P. notoginseng* remain poorly characterized. In this study, we isolated an antagonistic fungal strain identified as *T. asperellum* Ta1 from the rhizosphere soil of healthy *P. notoginseng* plants cultivated in an area with a high incidence of root rot. Our objectives were to investigate its antifungal activity, assess its growth-promoting effects on *P. notoginseng*, confirm its biocontrol efficacy against *P. notoginseng* root rot, and elucidate the mechanisms by which *T. asperellum* Ta1 promotes plant growth and enhances defense responses against *F. solani* infection through metabolomic and transcriptomic analyses.

## 2. Materials and Methods

### 2.1. Plant and Fungal Materials

Healthy two-year-old *P. notoginseng* plants lacking root rot symptoms, such as leaf yellowing, root softening, and blackening, were sourced from a cultivation base in Qiubei County, Wenshan Zhuang and Miao Autonomous Prefecture, Yunnan Province (latitude 23°49′16.99″ N, longitude 104°06′12.99″ E). Five root rot pathogens (*F. solani*, *F. oxysporum*, *Fusarium graminearum*, *Alternaria alternata*, and *Fusarium equiseti*) were tested in this study. Roots of *P. notoginseng* exhibiting symptoms of root rot were used to isolate the pathogens, which were subsequently identified and preserved at −80 °C refrigerator using methods published by Zhou et al. [21]. Prior to use, these fungi were cultured on potato dextrose agar (PDA) medium in a mold incubator at 28 °C for 7 days to activate the fungal strains.

### 2.2. Isolation and Identification of Antagonistic Strains

Rhizosphere soil (0.5 g) from a three-year-old healthy *P. notoginseng* plant was diluted with sterile distilled water. After centrifugation, the supernatant was inoculated onto PDA medium. Isolation of *Trichoderma* spp. strain was conducted according to the method described by Zhu et al. [22]. The antagonistic activity of the isolated strain was evaluated using the dual-culture method [23]. Each dual-culture assay was repeated three times. The root rot pathogens were co-cultured with the identified *Trichoderma* spp. strain on individual plates in a mold incubator at 28 °C for 7 days. The growth of the fungi was closely monitored, and the growth inhibition rate was calculated using the following formula: Inhibition rate (%) = (Colony diameter of control − Colony diameter of treatment)/Colony diameter of control × 100%. Multiple testing was used to analyze the statistical differences between different groups.

The strain demonstrating significant antifungal activity was selected for further assays. Genomic DNA was extracted using the CTAB method [24], and PCR amplification was conducted with internally transcribed spacer sequence 1/4 (ITS 1/4) primers and translation elongation factor 1 alpha (EF1-α) primers (Table S1) [25]. The PCR product was sequenced by Sangon Biotech Co., Ltd. (Shanghai, China). Homology analysis of the sequencing results was performed using NCBI BLAST (https://blast.ncbi.nlm.nih.gov/Blast.cgi, accessed on 25 November 2025 ), and a phylogenetic tree was constructed using Mega 7.0 to classify the isolated fungal strain in relation to known fungi.

### 2.3. Antifungal Analysis of T. asperellum Ta1 Fermentation Broth

*T. asperellum* Ta1 was inoculated into potato dextrose broth (PDB) and incubated at 28 °C with a shaking speed of 150 rpm for three days. The mycelia were then removed by filtering through sterile gauze. The resultant supernatant, referred to as Ta1 aseptic fermentation broth, was obtained by centrifuging the filtrate at 10,000 rpm for 20 min. Mycelia from *F. solani* or *F. oxysporum*, previously cultured on PDA at 28 °C for 7 days, were suspended in PDB to prepare a conidia suspension, adjusting the concentration to 1 × 10^6^ conidia/mL. In the experimental group, Ta1 fermentation broth (200 μL) was mixed with the conidia suspension of *F. solani* or *F. oxysporum* (200 μL, 1 × 10^5^ conidia/mL), respectively. The conidia suspension of *F. solani* or *F. oxysporum* treated with 200 μL of PDB medium served as the negative control. After culturing both the experimental and control groups in an incubator at 28 °C for 36 h, the germination status of the fungal conidia was observed using an inverted fluorescence microscope (Nikon, Tokyo, Japan). This antifungal assay of Ta1 fermentation broth was repeated three times.

Subsequently, the Ta1 fermentation broth was mixed with PDA medium at a 1:4 ratio, and then mycelia of *F. solani* or *F. oxysporum* were inoculated onto the medium. After incubation for 4 days at 28 °C, fungal growth was recorded using a camera (Leica, Wetzlar, Germany). Mycelia were collected, and total RNA was extracted. cDNA synthesis was performed using the GoTaq^®^ RT-qPCR system (Promega, Madison, WI, USA). qRT-PCR was conducted with specific primers targeting pathogenicity-related genes of *F. solani* or *F. oxysporum* (Table S1), employing *β-actin* of *F. solani* or *F. oxysporum* as the internal reference gene. The relative expression levels of the pathogenicity-related genes were calculated using the 2^−ΔΔCt^ method [26]. This experiment included three biological replicates and three technical replicates, and a *t*-test was used to determine statistical differences between the experimental and control groups.

### 2.4. Identification of Metabolites Secreted by T. asperellum Ta1

To identify metabolites secreted by *T. asperellum* Ta1, 20 μL of Ta1 fermentation broth was mixed with 120 μL of 50% methanol. After centrifugation, 10 μL of supernatant was obtained for metabolite detection using ultra-high-performance liquid chromatography tandem mass spectrometry (UPLC-MS/MS, Thermo, Waltham, MA, USA) at Lianchuan Biotechnology Co., Ltd. (Hangzhou, China). KEGG pathway enrichment analysis of metabolites was performed using the MBROLE 2.0 software (https://csbg.cnb.csic.es, accessed on 1 October 2023). A total of three sets of Ta1 fermentation broth were used for UPLC-MS/MS analysis.

### 2.5. Root Rot Control and Growth Promotion Analysis of T. asperellum Ta1 on P. notoginseng

A total of 36 two-year-old *P. notoginseng* plants were transplanted into 12 two-gallon pots filled with a mixture of humus soil and river sand (3:1, pH 5.5–6.5) and acclimatized under controlled environmental conditions (25 °C, 70% humidity, 12 h photoperiod) for 7 days. The control group consisted of 9 *P. notoginseng* plants (3 pots) that were treated with sterile water for 3 months. In the first experimental group, a total of 9 *P. notoginseng* plants were pretreated with *T. asperellum* Ta1 for 2 months (once every 2 weeks, 800 mL of Ta1 conidia suspension with a concentration of 1 × 10^6^ conidia/mL each time), followed by inoculation with *F. solani* for 28 days (once every 7 days, 800 mL of *F. solani* conidia suspension with a concentration of 1 × 10^6^ conidia/mL each time), designated as the Ta1Fs group. In the second experimental group, 9 *P. notoginseng* plants were treated with Ta1 (once every 2 weeks, 800 mL of Ta1 conidia suspension with a concentration of 1 × 10^6^ conidia/mL each time) for 2 months, followed by treatment with water for another 28 days, referred to as the Ta1 group. In the third experimental group, the 9 *P. notoginseng* plants were pretreated with sterile water for 2 months and then inoculated with *F. solani* for 28 days (once every 7 days, 800 mL of *F. solani* conidia suspension with a concentration of 1 × 10^6^ conidia/mL each time), designated as the Fs group. Each group included three biological replicates. The phenotypic traits of *P. notoginseng* were documented using a camera (Canon, Tokyo, Japan).

After the experiment was completed, *P. notoginseng* plants were collected and evaluated for root rot severity. The disease severity index (DSI) and control efficiency were calculated according to the methods described by Xiao et al. [27]. Additionally, the roots of 3 plants from each group were collected and combined as one biological sample. These roots were used to determine the biomass of *F. solani* in *P. notoginseng* using the loop-mediated isothermal amplification (LAMP) system established by our laboratory [28]. The recombinant vector of *FsDAO* (D-amino acid oxidase gene, pGEM-T-*DAO*, 0.9 ng/μL) was used as positive control, and sterile water was used as negative control. This experiment included three biological replicates and three technical replicates.

### 2.6. Determination of Growth and Agronomic Parameters of P. notoginseng

A total of 120 two-year-old *P. notoginseng* plants were utilized in the field experiment. The field experiment was conducted in Qiubei County, Wenshan Zhuang and Miao Autonomous Prefecture, Yunnan Province (latitude 23°49′16.99″ N, longitude 104°06′12.99″ E) in 2024. *T. asperellum* Ta1 was applied to irrigate the roots of 60 *P. notoginseng* plants for 6 months (once every 2 weeks, 400 mL of Ta1 conidia suspension with a concentration of 1 × 10^6^ conidia/mL per plant). The control group comprised another 60 *P. notoginseng* plants treated with water (400 mL each time per plant). The protocols of the field experiment were the same as the methods described by Gan et al. [10].

After 6 months, *P. notoginseng* plants were collected using a five-point sampling method, followed by measurements of various parameters, including plant height, bolting stem height, plant fresh weight, fresh and dry weight of roots, stem length, stem diameter, leaf length, and leaf width. Additionally, leaf samples from 3 *P. notoginseng* plants were collected and combined as one biological replicate to assess the contents of total chlorophyll, chlorophyll a, and chlorophyll b. Root samples were analyzed for total amino acids, total protein, fructose, and glucose content. The enzyme activities of fructose-1,6-diphosphate aldolase (FBP), fructose-1,6-diphosphatase (FBA), and ribulose-1,5-bisphosphate carboxylase/oxygenase (Rubisco) in the leaves of *P. notoginseng* were determined using methods described by Chen et al. [29]. Furthermore, the contents of plant endogenous hormones and five saponins (R1, Rg1, Re, Rb1, Rd) in roots were quantified based on the methods published by Gan et al. [10]. According to the transcriptome data published by Gan et al. [10], the expression of certain genes related to plant hormone biosynthesis in *P. notoginseng* changed following *B. subtilis* Pn1 treatment. Therefore, specific primers for these genes were designed (Table S2) and used for qRT-PCR analysis in this study, with *P. notoginseng β-actin* serving as the internal reference gene. This study included three biological replicates and three technical replicates. A *t*-test was employed to determine statistical differences between the experimental and control groups.

### 2.7. Transcriptome and Metabolome Analysis of P. notoginseng

To investigate the impact of *T. asperellum* Ta1 on the interaction between *P. notoginseng* and *F. solani*, two-year-old *P. notoginseng* plants were divided into four experimental groups: Fs, Ta1Fs, Ta1, and control, following the treatments and inoculations described in Section 2.5. Each group comprised 30 *P. notoginseng* plants. The roots of 10 *P. notoginseng* plants from each group were pooled to create one biological sample, and then 0.5 g root samples were collected from each group for transcriptome or proteome analysis. The methods for transcriptome sequencing and data analysis were the same as those reported by Deng et al. [30].

Total RNA from *P. notoginseng* roots was extracted using Trizol reagent (Invitrogen, Carlsbad, CA, USA), and the quantity and purity were analyzed using the Bioanalyzer 2100 and RNA 1000 Nano Lab Chip Kit (Agilent, Santa Clara, CA, USA). Following purification, the mRNA was fragmented into small pieces, and the cleaved RNA fragments were reverse-transcribed to construct the cDNA library according to the protocol of the mRNA Seq sample preparation kit (Illumina, San Diego, CA, USA). Paired-end sequencing was performed using an Illumina Novaseq™ 6000 (LC Sciences, Houston, TX, USA). Salmon version 0.8.2 was used to evaluate the expression levels of unigenes. Differentially expressed genes (DEGs) were selected for KEGG (Kyoto Encyclopedia of Genes and Genomes) enrichment analysis, considering a fold change ≥2 or ≤0.5 and a *p*-value ≤ 0.05 to identify DEGs. The aforementioned testing was completed by Lianchuan Biotech Company (Hangzhou, China).

For metabolomic analysis, 100 mg of root tissue was ground in liquid nitrogen, followed by the addition of 120 μL of 50% methanol for homogenization. After vigorous vortexing and centrifugation, the supernatant was collected and allowed to stand at room temperature (25 °C) for 10 min. The supernatant was then stored overnight at −20 °C and centrifuged at 4000× *g* for 20 min to yield metabolite samples for UPLC-MS/MS analysis. UPLC-MS/MS was performed using a Scientific UltiMate 3000 HPLC system (Thermo, Waltham, MA, USA), and an ACQUITY UPLC BEH C18 column (1.8 μm, 100 mm × 2.1 mm, Waters, Milford, MA, USA) was used for reversed-phase separation. A high-resolution tandem mass spectrometer, Triple TOF6600plus (SCIEX, Framingham, MA, USA), was utilized to detect the MS of each metabolite. The obtained MS data were processed to extract retention times and accurate masses. Metabolites were annotated by aligning the *m*/*z* of samples with entries in the KEGG database. Differentially expressed metabolites (DEMs) were classified based on phytochemical compounds in the KEGG database, and KEGG enrichment analysis of DEMs was conducted. The aforementioned testing was entrusted to Lianchuan Biotech Company (Hangzhou, China).

A Venn diagram analysis was performed using the OmicStudio platform (https://www.omicstudio.cn) [31] to integrate the transcriptomic and metabolomic data. In the comparison between Fs and control, the top 30 differentially expressed pathways (DEPs) from the transcriptome and metabolome were utilized to identify co-differentially expressed pathways (Co-DEPs) (Figure S1a). Co-DEPs for the other two comparisons (Fs vs. control and Ta1Fs vs. control) were identified using the same method (Figure S1b,c). Moreover, the DEMs and DEGs in Co-DEPs underwent advanced heatmap analysis employing the OmicStudio platform.

## 3. Results

### 3.1. Trichoderma asperellum Ta1 Inhibits Root Rot Pathogens

**Figure 1:**
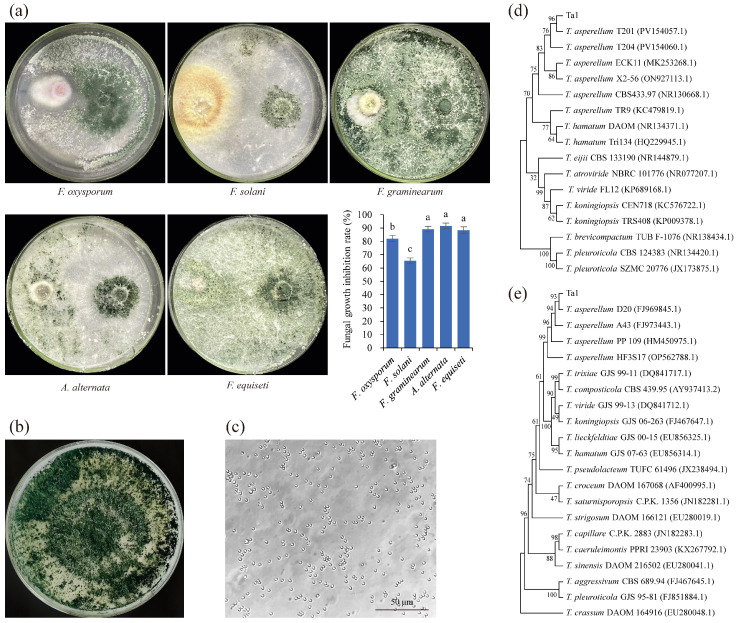
A strain capable of inhibiting root rot pathogens, isolated from the rhizosphere soil of healthy *Panax notoginseng*, was identified as *Trichoderma asperellum* Ta1. (**a**) The isolated antagonistic fungus inhibited the growth of *Fusarium oxysporum*, *Fusarium graminearum*, *Fusarium solani*, *Alternaria alternata*, and *Fusarium equiseti*; different lowercase letters indicated statistically significant differences between different samples (*p* < 0.05). (**b**) The mycelial morphology of the isolated antagonistic fungus. (**c**) The conidial morphology of the isolated antagonistic fungus. (**d**) Phylogenetic analysis based on ITS 1/4. (**e**) Phylogenetic analysis based on EF1-α. These experiments were replicated three times.

A *Trichoderma* spp. strain was isolated from the rhizosphere soil of healthy *P. notoginseng* plants, demonstrating significant inhibitory effects on various root rot pathogens (Figure 1a). Following dual culture with *Trichoderma* spp. strains on PDA plates, the growth of *F. oxysporum*, *F. graminearum*, *F. solani*, *A. alternata*, and *F. equiseti* was inhibited by 82.21%, 65.53%, 89.28%, 91.59%, and 88.45%, respectively. This indicates that the strain possesses broad-spectrum antifungal activity. Morphological observations revealed that the mycelia initially appeared white, with distinct concentric ring-shaped sporulation zones densely populated with conidia. The conidia transitioned in color from lighter shades on the periphery to darker hues toward the center, ultimately developing a dark green coloration. After 7 days of cultivation, the entire colony exhibited a dark green appearance due to extensive conidia production (Figure 1b). Microscopic examination confirmed that the spores of the isolated fungus were nearly circular (Figure 1c), a phenotype consistent with *T. asperellum*. Further analysis of ITS 1/4 (Sequence S1 (ITS sequence of Ta1)) showcased the highest homology with *T. asperellum*, sharing 99% sequence identity with *T. asperellum* T204, T201, and X2-56, respectively. The EF1-α sequence of the isolated *Trichoderma* spp. strain (Sequence S2, EF1-α sequence of Ta1) showed high homology with the EF1-α sequences of *T. asperellum* PP09, 10, and HF3S17. Phylogenetic analysis based on ITS1/4 or EF1-α sequences indicated that the isolated *Trichoderma* spp. strain formed a clade with *T. asperellum* T201 (PV154057.1) or *T. asperellum* PP09 (HM450975.1) (Figure 1d,e). These results confirmed that the isolated *Trichoderma* spp. strain belongs to *T. asperellum*; therefore, it was designated as Ta1.

### 3.2. T. asperellum Ta1 Fermentation Broth Inhibited Root Rot Pathogens

**Figure 2:**
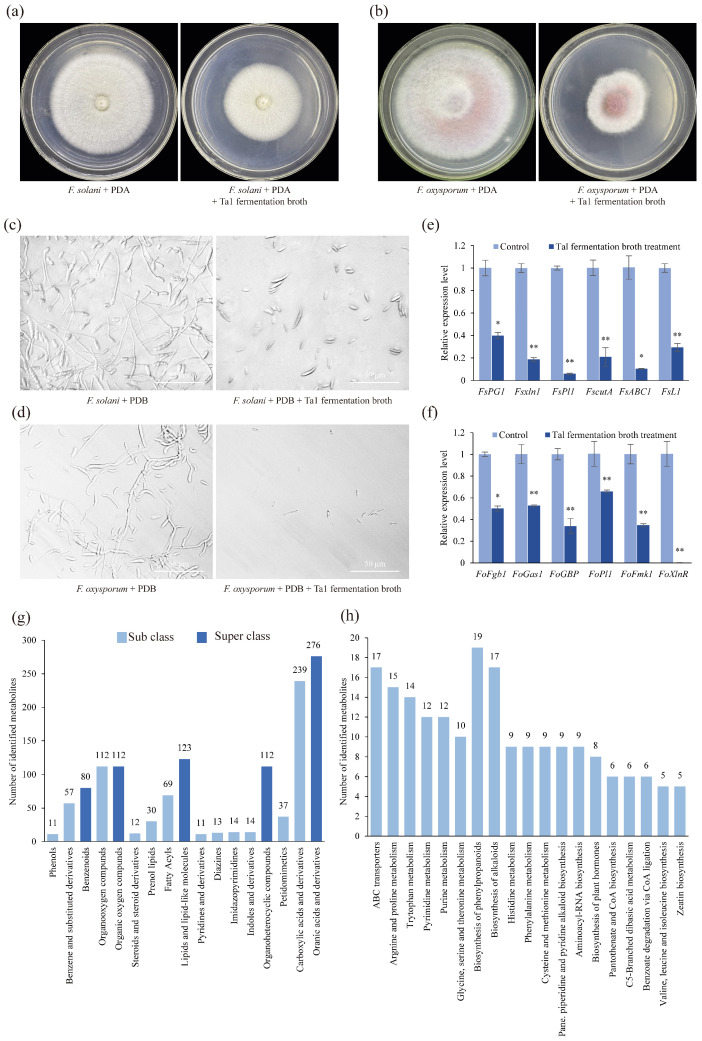
Antifungal and ingredient analysis of *T. asperellum* Ta1 fermentation broth. (**a**,**b**) The growth of *F. solani* and *F. oxysporum* was inhibited by Ta1 fermentation broth. (**c**,**d**) After treatment with Ta1 fermentation broth for 36 h, the conidial germination of *F. solani* and *F. oxysporum* was inhibited; these assays were repeated three times. (**e**,**f**) After Ta1 fermentation broth treatment, the expression of pathogenicity-related genes in *F. solani* and *F. oxysporum* was downregulated; Control, *F. solani* or *F. oxysporum* cultivated in PDB; there were three biological replicates and three technical replicates included in this experiment; *t*-test analysis was performed to assess significant differences between the Ta1 fermentation broth treatment and the control group (*, *p* < 0.05; **, *p* < 0.01). (**g**) Classification of metabolites from Ta1 fermentation broth. (**h**) KEGG enrichment analysis of metabolites from Ta1 fermentation broth.

After culturing on media containing Ta1 fermentation broth for 4 days, the growth of *F. solani* was significantly retarded, exhibiting a mycelial diameter of only 1.89 cm in the treatment group, compared to 3.72 cm in the control group—1.96 times larger than that of the treatment group (Figure 2a). Similar inhibitory effects on the growth of *F. oxysporum* were observed (Figure 2b). Furthermore, Ta1 fermentation broth inhibited the spore germination of both *F. solani* and *F. oxysporum*, with treated spores showing a marked decrease in germination, whereas control spores germinated vigorously (Figure 2c,d).

Additionally, the expression levels of pathogenicity-related genes in *F. solani* were significantly downregulated following treatment with Ta1 fermentation broth. After 4 days of treatment, the expression of genes such as *FsPG*, *Fsxln1*, and *FsPl1* was notably decreased (Figure 2e). The expression levels of *FsPG* (which is involved in plant cell wall degradation) in *F. solani* treated with Ta1 fermentation broth were 39.92% of those in the control. The transcriptional expression levels of *Fsxln1* (a transcriptional activator gene of the xylan decomposition system) and *FsPl1* (a pectin lyase gene) were significantly lower than those in the control group. The expression levels of *Fsxln1* and *FsPl1* were 18.68% and 5.86% of those in the control, respectively.

Similarly, after Ta1 fermentation broth treatment, the expression of several pathogenicity-related genes in *F. oxysporum* was decreased (Figure 2f). After treatment with Ta1 fermentation broth, the expression of *F. oxysporum* xylanase transcriptional activator (*FoXlnR*), which influences the growth of *F. oxysporum* in saprophytic and pathogenic stages, was 0.2% of that in the control. In addition, the expression of two virulence genes of *F. oxysporum*, mitogen-activated protein kinase (*FoFmk1*) and guanine nucleotide-binding protein (*FoFgb1*), was also decreased after treatment with Ta1 fermentation broth. The expression levels of two *F. oxysporum* genes related to plant cell wall degradation, beta-1,3-glucanosyltransferase (*FoGas1*) and pectate lyase (*FoPl1*), were 52.85% and 65.83% of those in the control, respectively. These findings indicate that the fermentation broth of *T. asperellum* Ta1 inhibits the root rot pathogens.

### 3.3. Identification of Metabolites in Ta1 Fermentation Broth via UPLC-MS/MS Analysis

To investigate the specific composition of metabolites in Ta1 fermentation broth, UPLC-MS/MS analysis was performed, identifying a total of 791 metabolites. In terms of superclass classification, the majority of metabolites identified were organic acids and their derivatives. Various antifungal metabolites, including organic acids (e.g., pipecolic acid), alkaloids (e.g., vincristine and bellaradine), and phenylpropanoids (e.g., phloretin and coumaric acid), were found in the Ta1 fermentation broth (Figure 2g). Furthermore, KEGG enrichment analysis revealed that these metabolites were primarily enriched in phenylpropanoids (e.g., chlorogenic acid and 2-coumaric acid), alkaloids from the shikimate metabolic pathway (e.g., physostigmine), and plant hormones (e.g., indoleacetic acid) (Figure 2h). Therefore, it can be concluded that Ta1 inhibits *F. solani* growth through the production of antifungal metabolites while synthesizing indoles and their derivatives, such as indoleacetic acid, to promote *P. notoginseng* growth.

### 3.4. T. asperellum Ta1 Effectively Controlled Root Rot in P. notoginseng

**Figure 3:**
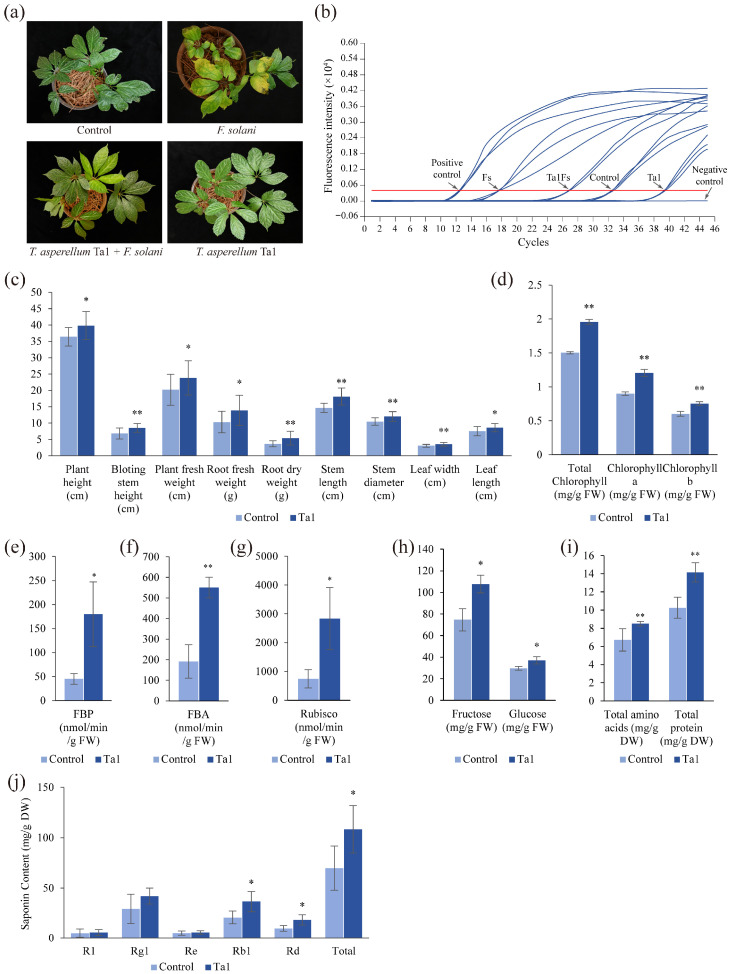
*T. asperellum* Ta1 suppressed the root rot caused by *F. solani* and promoted the growth of *P. notoginseng*. (**a**) *T. asperellum* Ta1 reduced the incidence of root rot in *P. notoginseng*; Control, sterile water; Ta1Fs, *T. asperellum* Ta1 treatment + *F. solani* inoculation; Ta1, only *T. asperellum* treatment; Fs, only *F. solani* inoculation. (**b**) The LAMP determination of *F. solani* in *P. notoginseng* plants. Positive control, plasmids of pGEM-T-*DAO*; Negative control, sterile water; Red line, the threshold of LAMP reaction. (**c**) Ta1 application influenced several agronomic traits of *P. notoginseng*. (**d**) The biosynthesis of total chlorophyll, chlorophyll a, and chlorophyll b in *P. notoginseng* was influenced by Ta1 treatment. (**e**–**g**) The activities of FBP, FBA, and Rubisco in *P. notoginseng* changed after Ta1 application. (**h**) The influence of Ta1 treatment on fructose and glucose levels. (**i**) The total amino acid and total protein contents in *P. notoginseng* changed following Ta1 application. (**j**) The influence of Ta1 treatment on saponin contents. All of these experiments contained three biological replicates. *t*-test analysis was performed to assess significant differences between treatment and control groups (*, *p* < 0.05; **, *p* < 0.01).

To evaluate the effectiveness of Ta1 in controlling root rot, a pot experiment was conducted involving four groups of two-year-old *P. notoginseng*. Following inoculation with *F. solani*, the resistance of *P. notoginseng* pretreated with Ta1 was found to be higher than that of the unpretreated Fs group (Figure 3a). The leaves of the Fs group exhibited yellowing and wilting, contrasting with the green leaves of the Ta1Fs group. Additionally, the control and Ta1 groups, which were not inoculated with *F. solani*, displayed healthy growth. The results from the DSI calculations indicated that *P. notoginseng* in the Fs group had typical root rot symptoms, with a DSI of 89%. In contrast, the DSI of *P. notoginseng* in the Ta1Fs group was 36%, significantly lower than that of the Fs group. No root rot symptoms were detected in the control and Ta1 groups. Based on the DSI values of the Fs and Ta1Fs groups, the biocontrol efficiency of Ta1 was calculated to be 59.55%. Concurrently, LAMP results demonstrated that the biomass of *F. solani* in the Fs group was the highest (Figure 3b), correlating with the lowest Ct value. After Ta1 pretreatment, the biomass of *F. solani* in *P. notoginseng* (Ta1Fs group) was downregulated. Furthermore, only a small amount of *F. solani* was detected in the control and Ta1 groups, with the Ta1 group showing the lowest content of *F. solani* and the highest Ct value. Additionally, *P. notoginseng* plants in the Ta1 group exhibited enhanced growth vigor compared to those in the control group. The key agronomic traits, photosynthetic parameters, levels of primary metabolites, and the accumulation of bioactive saponins in *P. notoginseng* increased following Ta1 treatment (Figure 3c–j). These results indicate that Ta1 treatment effectively prevented the onset of root rot in *P. notoginseng* resulting from *F. solani* infection.

### 3.5. T. asperellum Ta1 Promoted Growth and Agronomic Trait Development in P. notoginseng

To investigate the impact of *T. asperellum* Ta1 on the growth and agronomic traits of *P. notoginseng*, several parameters were assessed. Following Ta1 treatment, significant increases were observed in plant height, bolting stem height, plant fresh weight, root fresh and dry weight, stem length, stem diameter, and leaf dimensions (length and width) compared to the control group (Figure 3c). The plant height reached 39.82 cm following Ta1 treatment, indicating a 9% increase compared to the control group. Additionally, the plant weight was 23.8 g, which is 1.18 times higher than that of the control group, while fresh root weight demonstrated a remarkable 134% increase relative to controls.

Photosynthesis was notably enhanced in *P. notoginseng* after treatment with Ta1, as indicated by increased levels of photosynthetic pigments (total chlorophyll, chlorophyll a, and chlorophyll b) (Figure 3d). The activities of photosynthesis-related enzymes (FBP, FBA, and Rubisco) were also significantly elevated (Figure 3e–g), with Rubisco activity showing the most substantial increase—3.8 times that of the control. Likewise, the synthesis of fructose, glucose, total amino acids, and total protein increased post-Ta1 treatment, with elevations of 1.44-, 1.25-, 1.26-, and 1.37-fold compared to the control, respectively (Figure 3h,i).

Additionally, the biosynthesis of saponins (the primary active ingredients in *P. notoginseng*) was significantly upregulated following Ta1 treatment (Figure 3j). The contents of R1, Rg1, Re, Rb1, Rd, and total saponins (the sum of five monomeric saponins) in *P. notoginseng* increased, with total saponins showing a 1.55-fold increase compared to the control group. These results demonstrate that Ta1 treatment enhanced the growth and development of agronomic traits by improving photosynthesis and the accumulation of carbohydrates and amino acids.

### 3.6. Ta1 Enhanced Biosynthesis of Growth-Promoting Hormones in P. notoginseng

**Figure 4:**
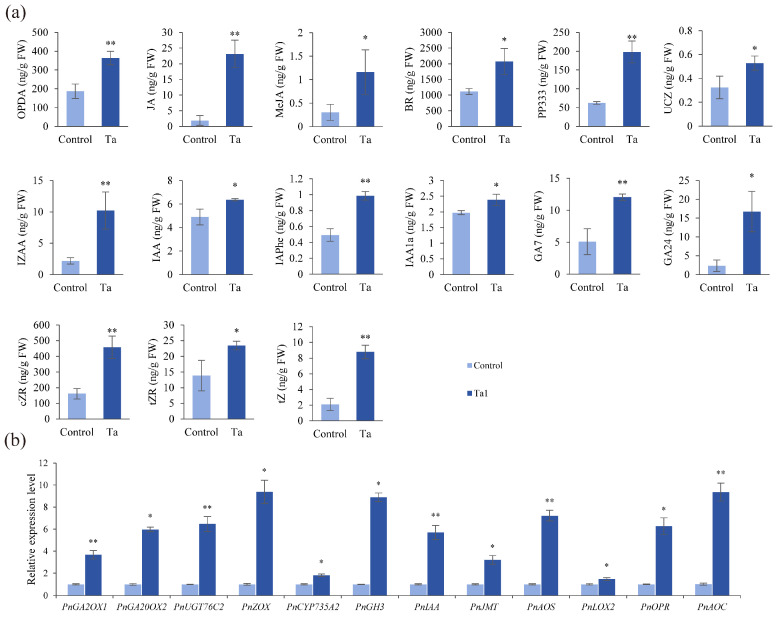
*T. asperellum* Ta1 enhanced the biosynthesis of defense-related and growth-promoting hormones in *P. notoginseng*. (**a**) Ta1 treatment increased the contents of defense-related and growth-promoting hormones. (**b**) Ta1 treatment upregulated the expression of genes related to hormone biosynthesis. There were three biological replicates and three technical replicates included in these experiments; *t*-test analysis was performed to assess significant differences between Ta1 treatment and control groups (*, *p* < 0.05; **, *p* < 0.01).

Phytohormones are critical regulators of plant development. To further explore the mechanisms underlying Ta1’s promotion of *P. notoginseng* growth, levels of various phytohormones were quantified using UPLC-MS/MS (Figure 4a). The results indicated that the accumulation of plant defense-related signaling molecules, including 12-oxophytodienoic acid (OPDA), jasmonic acid (JA), and methyl jasmonate (MeJA), was significantly increased by 1.9-, 12.1-, and 3.8-fold compared to the control, respectively. Additionally, levels of plant growth-related hormones, such as indole-3-acetic acid (IAA, IAAla, IZAA, IAPhe), gibberellins (GA24, GA7), and zeatin (tZ, tZR, cZR), markedly increased following Ta1 treatment. Among these hormones, tZ exhibited the most significant change, reaching 8.78 ng/g, a 4.17-fold increase over the control.

Furthermore, gene expression analysis related to phytohormone biosynthesis was conducted via qPCR following Ta1 treatment (Figure 4b). The gibberellin biosynthesis genes, including *GA2OX1* and *GA20OX2*, were found to be upregulated. The most significant upregulation was observed in ZOX, with a 9.38-fold increase. Additionally, expression of jasmonic acid signaling-related genes (*JMT*, *AOS*, *LOX2*, *OPR*, and *AOC*) also showed upregulation. These results collectively indicate that the exogenous application of Ta1 promotes the biosynthesis of growth-promoting hormones in *P. notoginseng*.

### 3.7. Transcriptomics Profiling of P. notoginseng with Ta1 Treatment Against F. solani Infection

**Figure 5:**
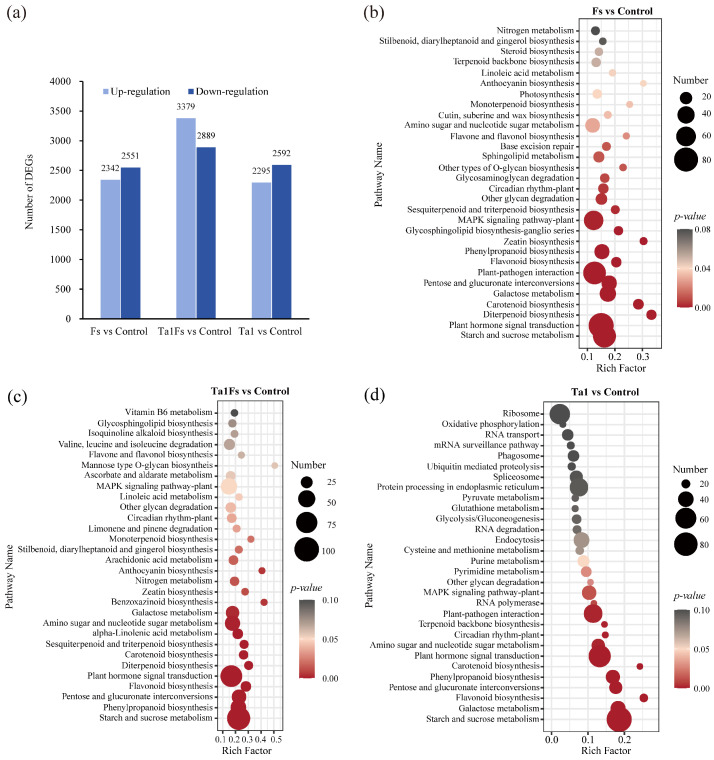
Transcriptome analysis of *P. notoginseng* with Ta1 treatment during *F. solani* infection. (**a**) Number of differentially expressed genes (DEGs) in Fs vs. control, Ta1Fs vs. control, and Ta1 vs. control. (**b**) KEGG enrichment analysis of DEGs for Fs vs. control. (**c**) KEGG enrichment analysis of DEGs for Ta1Fs vs. control. (**d**) KEGG enrichment analysis of DEGs for Ta1 vs. control.

Transcriptome analysis uncovered significant differential gene expression in *P. notoginseng* following different treatments. When compared to the control group, *F. solani* infection led to the significant upregulation of 2342 unigenes and the downregulation of 2551 unigenes in the Fs group. In *P. notoginseng* pretreated with *T. asperellum* Ta1 prior to *F. solani* inoculation (Ta1Fs group), 3379 unigenes were upregulated, and 2889 unigenes were downregulated. Treatment with Ta1 alone (Ta1 group) resulted in 2295 upregulated and 2592 downregulated unigenes (Figure 5a). Subsequently, all differentially expressed genes (DEGs) were analyzed for KEGG pathway enrichment.

The 85 DEGs in the Fs group were enriched in the plant hormone signal transduction pathway; moreover, 75 DEGs were associated with starch and sucrose metabolism, and 74 DEGs were related to the plant–pathogen interaction pathway (Figure 5b). The DEGs of the Ta1Fs group were significantly enriched in pathways related to starch and sucrose metabolism (104 DEGs), plant hormone signal transduction (95 DEGs), phenylpropanoid biosynthesis (58 DEGs), and flavonoid biosynthesis (27 DEGs) (Figure 5c). Furthermore, the DEGs of the Ta1 group were primarily related to starch and sucrose metabolism (86 DEGs), plant hormone signal transduction (75 DEGs), and the plant–pathogen interaction pathway (63 DEGs) (Figure 5d). The KEGG enrichment analysis demonstrated that the application of Ta1 significantly regulated the *P. notoginseng* defense response against *F. solani* infection, as evidenced by changes in the expression of phenylpropanoid and flavonoid biosynthesis pathways as well as the plant hormone signal transduction pathways.

### 3.8. Metabolomics Profiling of P. notoginseng with Ta1 Treatment Against F. solani Infection

**Figure 6:**
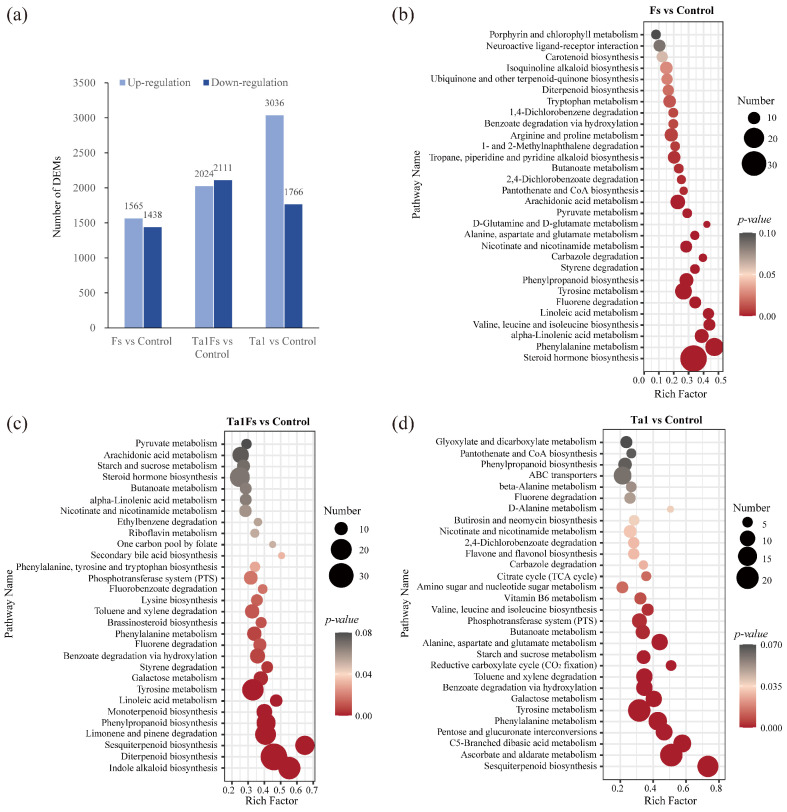
Metabolome analysis of *P. notoginseng* with Ta1 treatment during *F. solani* infection. (**a**) Number of differentially expressed metabolites (DEMs) in Fs vs. control, Ta1Fs vs. control, and Ta1 vs. control. (**b**) KEGG enrichment analysis of DEMs for Fs vs. control. (**c**) KEGG enrichment analysis of DEMs for Ta1Fs vs. control. (**d**) KEGG enrichment analysis of DEMs for Ta1 vs. control.

Through metabolome analysis, a total of 3003 metabolites were differentially accumulated in the Fs group. The abundance of 1565 metabolites was upregulated, while 1438 metabolites were downregulated when compared to the control. In the Ta1Fs group, a total of 4135 metabolites were differentially expressed, with 2024 metabolites upregulated and 2111 metabolites downregulated. Additionally, there were 4802 differentially expressed metabolites (DEMs) in the Ta1 group, among which the abundance of 3036 metabolites increased and 1766 metabolites decreased (Figure 6a).

The KEGG enrichment analysis of DEMs in the Fs group revealed that these metabolites were mainly enriched in steroid hormone biosynthesis (33 DEMs), phenylalanine metabolism (21 DEMs), alpha-linolenic acid metabolism (15 DEMs), and valine, leucine, and isoleucine biosynthesis (12 DEMs) (Figure 6b). Similarly, we conducted KEGG enrichment analysis of DEMs in the Ta1Fs group, and the results were as follows: a total of 22 DEMs were enriched in indole alkaloid biosynthesis, 33 DEMs were involved in diterpenoid biosynthesis, 19 DEMs belonged to sesquiterpenoid biosynthesis, and 24 DEMs participated in limonene and pinene degradation (Figure 6c). The metabolomics profiling indicated some differences between the Fs group and the Ta1Fs group, and the differential accumulation of indole alkaloids, diterpenoids, and sesquiterpenoids may be important in the application of Ta1, which slowed the incidence of root rot in *P. notoginseng*.

In addition, the metabolomics profiling of the Ta1 group showed that the DEMs were mainly enriched in ascorbate/aldarate metabolism (24 DEMs) and pentose/glucuronate interconversions (24 DEMs) (Figure 6d). At the same time, many DEMs were involved in pathways related to amino sugar/nucleotide sugar metabolism (20 DEMs), starch/sucrose metabolism (16 DEMs), and galactose metabolism (16 DEMs). Furthermore, some amino acid-related metabolic pathways, including C5-branched dibasic acid metabolism (18 DEMs), phenylalanine metabolism (19 DEMs), tyrosine metabolism (24 DEMs), and valine/leucine/isoleucine biosynthesis (10 DEMs), also exhibited differential accumulation patterns. These results confirmed that Ta1 promotes the growth of *P. notoginseng* by enhancing the biosynthesis of carbohydrates and amino acids.

### 3.9. Combined Analysis of Transcriptome and Metabolome Reveals the Biocontrol and Growth-Promoting Mechanisms of Ta1 in P. notoginseng

**Figure 7:**
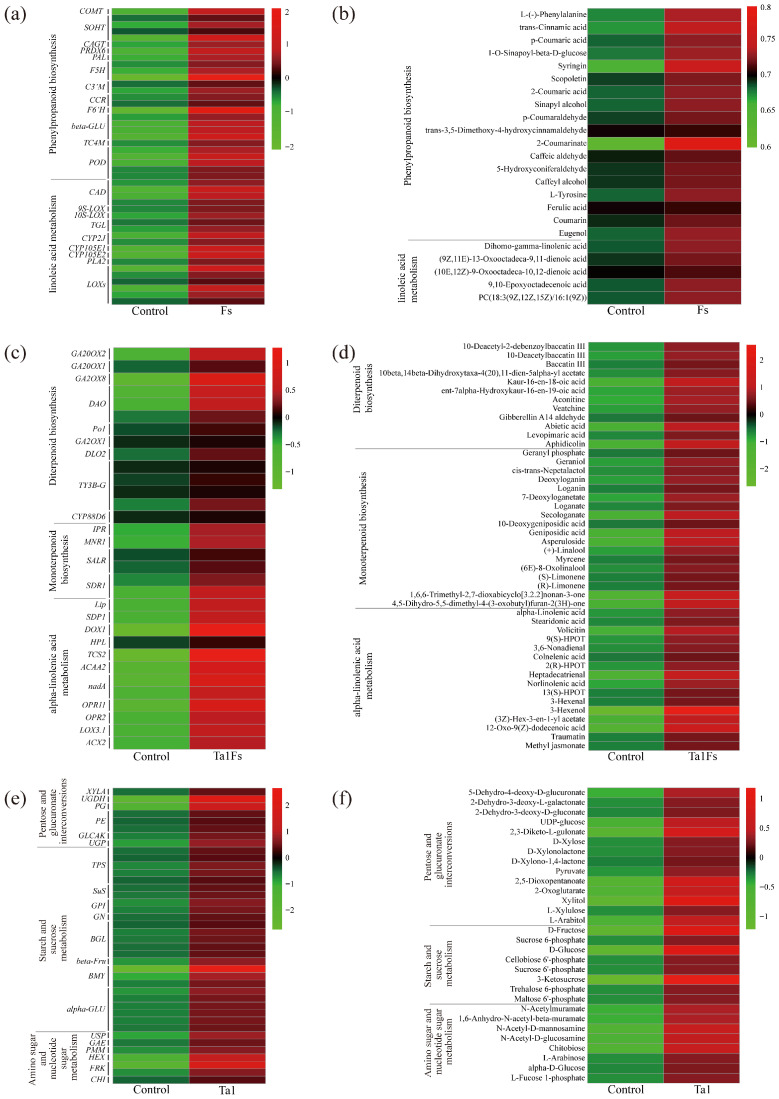
Heatmap of co-differentially expressed pathways (Co-DEPs) based on the integrated analysis of DEGs and DEMs. (**a**,**b**) Heatmap of Co-DEPs from the group Fs vs. control. (**c**,**d**) Heatmap of Co-DEPs from the group Ta1Fs vs. control. (**e**,**f**) Heatmap of Co-DEPs from the group Ta1 vs. control.

To analyze the changes in metabolomics and transcriptomics profiles of *P. notoginseng*, a Venn diagram was constructed based on the differentially expressed proteins (DEPs) from the comparison between the Fs group and the control. The results showed that the phenylpropanoid biosynthesis and linoleic acid metabolism pathways were co-DEPs in the Fs group (Table 1). The differentially expressed genes (DEGs) and DEMs related to phenylpropanoid biosynthesis showed significant changes compared with the control. The expression levels of the *COMT*, *SOHT*, and *CCR* genes were higher than those in the control (Figure 7a). The expression of *9S-LOX* and *CYP2J* was also upregulated after infection. In addition, the ion intensities of syringin, L-(-)-phenylalanine, and coumarin were 8.0, 5.2, and 5.0-fold higher than those of the control (Figure 7b). These results indicate that the infection by *F. solani* affects the biosynthesis of phenylpropanoids, thereby altering the defense response of *P. notoginseng*.

In the Ta1Fs group vs. control, a total of 8 co-DEPs were identified (Table 1). Among them, the synthesis of defense metabolites (e.g., diterpenoid biosynthesis and monoterpenoid biosynthesis), the plant hormone signaling molecule JA biosynthesis-related pathway (e.g., alpha-linolenic acid metabolism), and plant growth regulation pathways (e.g., limonene and pinene degradation, galactose metabolism, and starch and sucrose metabolism) were only co-differentially expressed in the Ta1Fs group. Therefore, through the analysis of DEMs and DEGs related to alpha-linolenic acid metabolism and diterpenoid and monoterpenoid biosynthesis, we found that the expression of genes related to diterpenoid biosynthesis, such as *GA20OX1*, *GA20OX2*, and *GA2OX8*, was upregulated by 3.0, 2.4, and 5.6-fold, respectively (Figure 7c). Diterpenoid compounds, such as aphidicolin, abietic acid, and 10-deacetylbaccatin III, significantly increased, with increases of 4.6, 4.5, and 3.0-fold in the Ta1Fs group compared to the control (Figure 7d). In addition, JA biosynthesis-related genes (e.g., *OPR*, *ACX*) were also upregulated. The contents of JA-related metabolites (12-Oxo-9(Z)-dodecenoic acid and methyl jasmonate) also increased. These results show that pretreatment with Ta1 enhanced the biosynthesis of diterpenoids/monoterpenoids and activated the JA signaling pathway in *P. notoginseng*, thereby boosting the defense response when threatened by *F. solani*.

For the comparison of Ta1 vs. control, some primary metabolic pathways related to pentose and glucuronate interconversions, starch and sucrose metabolism, and amino sugar and nucleotide sugar metabolism were co-differentially expressed (Table 1). The expression of genes involved in starch and sucrose biosynthesis, such as *BMY* and *TPS*, was significantly upregulated after Ta1 treatment (Figure 7e). Additionally, the expression levels of amino sugar and nucleotide sugar biosynthesis-related genes (*HEX* and *USP*) increased by 6.0- and 3.4-fold compared to the control. Moreover, the contents of some carbohydrates (such as UDP-glucose, D-fructose, and chitobiose) also increased in the *P. notoginseng* that underwent Ta1 treatment (Figure 7f). These results further revealed that Ta1 promotes the growth of *P. notoginseng* by enhancing the biosynthesis of carbohydrates and amino acids.

## 4. Discussion

The most significant challenge affecting the yield and quality of *P. notoginseng* is root rot, primarily caused by fungal pathogens such as *F. solani*. Currently, chemical methods remain the predominant approach for controlling root rot in *P. notoginseng* [32]. However, reliance on chemical treatments leads not only to environmental pollution but also to harmful chemical residues. In contrast, biological control methods have gained increasing attention as sustainable alternatives [5]. Among various biocontrol agents, *Trichoderma* spp. are highly regarded for their efficacy [33]. For instance, the strain *T. harzianum* QT20045 has shown significant antagonistic effects against *Sclerotium rolfsii*, aiding in the management of peanut stem rot [16]. Similarly, *T. hamatum* T382 demonstrated resistance to *Xanthomonas euvesicatoria*, which causes bacterial spot disease in tomato [34,35]. In this study, *T. asperellum* Ta1, isolated from the rhizosphere soil of healthy *P. notoginseng*, exhibited broad-spectrum antifungal activity against multiple pathogens, including *F. oxysporum*, *F. graminearum*, *F. solani*, *A. alternata*, and *F. equiseti* in vitro (Figure 1a). The fermentation broth of Ta1 inhibited the germination of *F. solani* spores and influenced the expression of pathogenicity-related genes (Figure 2). Furthermore, Ta1 effectively reduced the incidence of root rot in *P. notoginseng* (Figure 3a,b). In addition, Ta1 enhanced the biosynthesis of diterpenoids and monoterpenoids, as well as jasmonic acid biosynthesis in *P. notoginseng*, indirectly increasing the plant’s resistance to root rot (Figure 7c,d). These findings robustly indicate that *T. asperellum* Ta1 possesses significant biocontrol potential and could be instrumental in managing root rot in *P. notoginseng* or other plants susceptible to this disease, such as *Arachis hypogaea*, *Phalaenopsis amabilis*, and *Clivia miniata*.

Previous studies have noted that *T. atroviride* inhibits pathogens by producing a variety of metabolites, including peptaibols, steroids, anthraquinones, lactones, and monoterpenes, all of which exhibit antifungal activity [36]. Sterols extracted from *T. harzianum* and *Trichoderma koningii* have demonstrated strong antifungal properties against *Rhizoctonia solani*, *S. rolfsii*, *Macrophomina phaseolina*, and *F. oxysporum* [37]. For example, treating *Fusarium culmorum* mycelium with a crude extract of *T. harzianum* dissolved in ethanol resulted in a significant reduction in mycelial growth [38]. In contrast, no considerable difference was observed when the crude extract was dissolved in water. This study identifies metabolites from the fermentation broth of *T. asperellum* Ta1, predominantly comprising phenylpropanoids and alkaloids from the shikimate pathway (such as physostigmine) (Figure 2g,h). Meanwhile, Ta1 fermentation broth inhibited mycelial growth and spore germination of *F. solani* (Figure 2a–f). These results suggest that Ta1 produces various antifungal metabolites in its fermentation broth, warranting further investigation into their specific mechanisms of action against root rot pathogens. In particular, molecules with potential antifungal properties should be extracted and identified from Ta1 fermentation broth, and their effects on root rot pathogens merit further investigation.

The interaction between antagonistic fungi and plant defense mechanisms significantly enhances plant resistance to pathogens. For instance, *T. harzianum* T22 has been reported to improve the resistance of *Solanum lycopersicum* against *Cucumber mosaic virus* (CMV) by modulating jasmonic acid (JA), ethylene, and salicylic acid signaling. Following treatment with T22, the levels of these phytohormones increased, correlating with reduced CMV accumulation [39]. Similarly, *L. esculentum* grown with *T. asperellum* exhibited heightened resistance to infections from *Botrytis cinerea* and *F. oxysporum*, along with reduced levels of reactive oxygen species during infections [40]. *T. harzianum* has also enhanced grape defense responses to downy mildew by upregulating the activity of defense-related enzymes, including PAL, POD, and 1,3-glucanase, while also promoting the biosynthesis of lignin, callose, and hydrogen peroxide [41]. In this study, MeJA content in *P. notoginseng* significantly increased following Ta1 treatment (Figure 4). Additionally, the integrative analysis of transcriptomics and metabolomics indicated that pretreatment with Ta1 promoted the biosynthesis of diterpenoids and monoterpenoids, along with jasmonic acid biosynthesis in response to *F. solani* infection (Figure 7c,d). It is evident that *T. asperellum* Ta1 acts as a key antagonistic fungus by enhancing the accumulation of monoterpenes and diterpenes while activating JA signaling in *P. notoginseng*, thereby strengthening the plant’s immune response against root rot.

The growth-promoting effects of *Trichoderma* spp. contribute significantly to their widespread application in agriculture. For instance, treatment with a conidia suspension of *Trichoderma breve* Z2-03 for 10 days resulted in increased stem length, root length, stem fresh weight, root fresh weight, and biomass of rice compared to the control group [42]. A mixture of spores from *T. harzianum* (T18), *T. asperellum* (T9), *T. atroviride* (T14), and *T. hamatum* (T19) enhanced germination rates and improved seedling growth in flowering cabbage [43]. *T. harzianum* strains have been shown to produce indole-3-acetic acid (IAA), promoting tomato growth and controlling tomato wilt disease [44]. Moreover, treatment with *T. breve* Z2-03 increased IAA and chlorophyll contents in rice, further promoting plant growth [42]. In *Arabidopsis thaliana*, IAA synthesized by *Trichoderma virens* GV29.8 and *T. atroviride* IMI206040 contributed to an increase in lateral root yield [35, 45]. In this study, Ta1 treatment led to significant improvements in plant height, root length, stem length, and saponin content of *P. notoginseng* (Figure 3). The chlorophyll content and the activity of photosynthetic enzymes were markedly elevated (Figure 3c–g). Furthermore, the accumulation of plant growth-related hormones, including gibberellins, cytokinins, and IAA, was significantly enhanced (Figure 4a). Non-targeted metabolomic analyses indicated that plant growth-related hormones, such as IAA, were produced by Ta1 (Figure 2g,h). Integrative analysis of transcriptomics and metabolomics revealed that Ta1 treatment upregulated pathways associated with carbohydrate and amino acid biosynthesis (Figure 7e,f). Additionally, the growth of pathogenic fungi in the rhizosphere soil of *P. notoginseng* is inhibited following Ta1 treatment, thereby reducing stress from root rot. Conversely, Ta1 application promotes the biosynthesis of growth-related hormones, including IAA in *P. notoginseng*. It is evident that the application of Ta1 not only alleviates root rot stress but also enhances growth hormone secretion, both of which are beneficial for promoting the growth of *P. notoginseng* plants.

## 5. Conclusions

**Figure 8:**
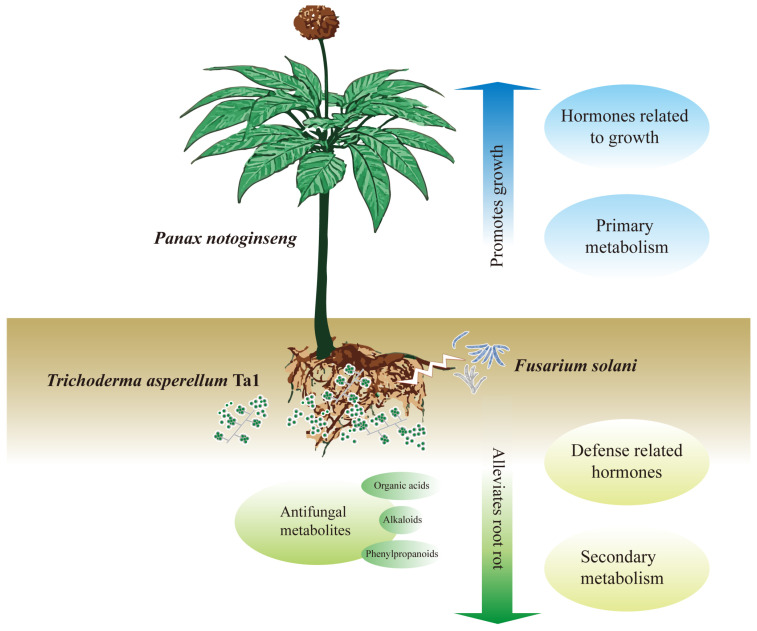
The exogenous application of *T. asperellum* Ta1 inhibited the growth and pathogenicity of *F. solani*, promoted the biosynthesis of diterpenoids and monoterpenoids, and enhanced the phenylpropanoid and linoleic acid metabolism, thus slowing down the incidence of root rot. Moreover, *T. asperellum* Ta1 promoted the growth of *P. notoginseng* by improving photosynthesis and increasing the contents of carbohydrates, amino acids, and growth-promoting hormones. Given its dual functions in disease control and growth promotion, Ta1 represents a promising biological control agent with great potential for application in *P. notoginseng* cultivation.

*T. asperellum* Ta1, isolated from the rhizosphere soil of *P. notoginseng*, effectively inhibits the growth of root rot pathogens. Additionally, the fermentation broth of Ta1 contains metabolites, including organic acids, alkaloids, and phenylpropanoids, that demonstrate significant antifungal activity. The application of Ta1 confers enhanced resistance to root rot in *P. notoginseng*, indicating that Ta1 has considerable potential as a green biological agent for controlling root rot, thereby reducing the use of chemical pesticides and maintaining the ecological balance of soil. Moreover, Ta1 promotes the growth of *P. notoginseng* by improving photosynthesis and enhancing the biosynthesis of carbohydrates, amino acids, and growth-promoting hormones. Consequently, the plant height, root weight, and saponin content of *P. notoginseng* increased, further enhancing its value. Integrative analysis of transcriptomic and metabolomic data revealed that Ta1 application elevates the biosynthesis of phenylpropanoids, diterpenoids, and monoterpenoids while interacting with jasmonic acid signaling pathways. Given its effectiveness in controlling root rot and promoting plant growth, *T. asperellum* Ta1 holds promise as a multifunctional agricultural probiotic for future applications (Figure 8).

## Figures and Tables

**Table 1:** Co-differentially expressed pathways of three compared groups.

Group	Co-Differentially Expressed Pathways Name
Fs vs. Control	Linoleic acid metabolism
	Phenylpropanoid biosynthesis
Ta1Fs vs. Control	Diterpenoid biosynthesis
	Limonene and pinene degradation
	Phenylpropanoid biosynthesis
	Monoterpenoid biosynthesis
	Linoleic acid metabolism
	Galactose metabolism
	alpha-Linolenic acid metabolism
	Starch and sucrose metabolism
Ta1 vs. Control	Pentose and glucuronate interconversions
	Galactose metabolism
	Starch and sucrose metabolism
	Amino sugar and nucleotide sugar metabolism
	Flavone and flavonol biosynthesis

## Data Availability

The original contributions presented in the study are included in the article/Supplementary Materials. Further inquiries can be directed to the corresponding author.

## Supplementary Materials

The following are available online at https://www.mdpi.com/article/10.3390/jof11120879/s1, Table S1: Specific primers for detecting fungal gene expression levels; Table S2: Specific primers used to detect the expression level of plant endogenous hormone biosynthesis-related genes. Figure S1: Schematic diagram of method for the multi-omics analysis. (a) The group Fs vs. Control. (b) The group Ta1Fs vs. Control. (c) The group Ta1 vs. Control; Sequence S1: ITS1/4 sequence of Ta1. Sequence S2: EF1-α sequence of Ta1.
