# Supplementary material for: Trichoderma asperellum Ta1 Alleviates Root Rot Caused by Fusarium solani and Promotes the Growth of Panax notoginseng
Source: J Fungi (Basel). 2025 Dec 11;11(12):879. doi: 10.3390/jof11120879 (PMC12733628; doi:10.3390/jof11120879)
Supplement: Supplementary file 1 [file jof-11-00879-s001.zip › jof-4001074-supplementary.pdf]

Table S1 Specific primers for fungi

Primer name	Primer sequence	Purpose
ITS	Forward: 5’ TCCGTAGGTGAACCTGCGG 3’<br>Reverse: 5’ TCCTCCGCTTATTGATATGC 3’	Cloning ITS 1/4 sequence from isolated *Trichoderma* spp.
EF1-α	Forward: 5’ CATCGAGAAGTTCGAGAAGG 3’<br>Reverse: 5’ TACTTGAAGGAACCCTTACC 3’	Cloning *EF1-α* sequence from isolated *Trichoderma* spp.
FsActin	Forward: 5’ TGATTGGTATGGGTCAGAAGGA 3’<br>Reverse: 5’ GGTGAGCAGGACGGGGT 3’	Detecting the expression level of *Fusarium solani*'s pathogenicity-related genes
FsPG1	Forward: 5’ ACCACTACTGGCGTAACCTGTG 3’<br>Reverse: 5’ TGATGCCTGCTGCCTTCATA 3’	
Fsxln1	Forward: 5’ GTTATTTCCAGTATTTCCTTCCG 3’<br>Reverse: 5’ TAGCGTTCCCAGCGTTGT 3’	
FsPl1	Forward: 5’ GCGGCATCAACACCAACTAC 3’<br>Reverse: 5’ TCAGGGCCAGAGCCAATC 3’	
FscutA	Forward: 5’ GTGCCCTGACGCGACTTT 3’<br>Reverse: 5’ GATTCGGCCACGGTTCTGTA 3’	
FsABC1	Forward: 5’ TCACTTGGAAACCAGCACCG 3’<br>Reverse: 5’ ACGAACAGCAGCAGAGGAGG 3’	
FsL1	Forward: 5’ GGAAACGACTTCTTCGCCAA 3’<br>Reverse: 5’ AGACCTACCGTGCCACCATT 3’	
FoActin	Forward: 5’ GAGACATTCAACGCCCCAG 3’<br>Reverse: 5’ GGTAAGATCACGACCAGCCA 3’	Detecting the expression level of *Fusarium oxysporum*'s pathogenicity-related genes
FoFgb1	Forward: 5’ TAACACATACCCCTACCGACG 3’<br>Reverse: 5’ CCACCCATTACTAACAGCCC 3’	
FoGas1	Forward: 5’ CTTTGTCCCGTGGTTGGTT 3’<br>Reverse: 5’ GCGGCTGTGTTGTTGTAGTG 3’	
FoGBP	Forward: 5’ ATCACATCTTGGTTTGG 3’<br>Reverse: 5’ GAACCGCCTACACGGTGA 3’	
FoPl1	Forward: 5’ AACTGGTGAGAAGGATGCTATGT 3’<br>Reverse: 5’ CTGGTCTGCTTGAGGGTGAT 3’	
FoFmk1	Forward: 5’ CCAACCTCCTCCTCAACGC 3’<br>Reverse: 5’ CCGACCACACATCAATAGCC 3’	
FoXlnR	Forward: 5’ CGAACCCACAATACCCTTACTG 3’<br>Reverse: 5’ TGATGGCTTTGCCCGAG 3’	

Table S2 Specific primers used to detect the expression level of plant endogenous hormone biosynthesis related genes

Primer name	Primer sequence
PnGA2OX1	Forward: 5’ AAGAGTGTAAGGCATAGGGT 3’<br>Reverse: 5’ GTAGTGATGGCAATGGAGC 3’
PnGA20OX2	Forward: 5’ GCCTCATTGTGATCCAACA 3’<br>Reverse: 5’ ACCACCTTATCCTCCTTCG 3’
PnUGT76C2	Forward: 5’ CTTCCGTACTTCCTCGCAT 3’<br>Reverse: 5’ CTCATTTACATTTTTCCCCC 3’
PnZOX	Forward: 5’ TAATCCTGTCTCCGTGTCCC 3’<br>Reverse: 5’ CATCTCCTTTATCAGCATCTCTC 3’
PnCYP735A2	Forward: 5’ CCGGCGTTTTATTGTTATG 3’<br>Reverse: 5’ GTATGCGTCTTGGGGTTAG 3’
PnGH3	Forward: 5’ GTGACACGAAAACCATACCG 3’<br>Reverse: 5’ ATTAATTGACGCACCCCTA 3’
PnIAA	Forward: 5’ GCTGCCATTCCTGCTAAAC 3’<br>Reverse: 5’ TACTTCCAAGTCGGCCTAT 3’
PnJMT	Forward: 5’ ATCATGTTGACAGAGGCAAT 3’<br>Reverse: 5’ ATGGTGGCTAATCATCGACT 3’
PnAOS	Forward: 5’ AATGGCGGCGATGGAA 3’<br>Reverse: 5’ ACGGTTGGAACCCGAATAA 3’
PnLOX2	Forward: 5’ TGGGCAGAAACTCCAGTTATTAATG 3’<br>Reverse: 5’ CCTTACTCAAAATGAGATTTGTGCC 3’
PnOPR	Forward: 5’ GTTACGACAGGGAAGATGGCAATAA 3’<br>Reverse: 5’ ACAAAATGGCAAGTGCATAAGTCAG 3’
PnAOC	Forward: 5’ AGCCCTATGTATCTCAGATTGGGTC 3’<br>Reverse: 5’ ACGTAAGCACCCTGTACGGATATCT 3’
PnActin	Forward: 5’ TCCAAGGGTGAATATGATGAATCG 3’<br>Reverse: 5’ AACCTCTCCAAAGAGAATTTCTGAGT 3’

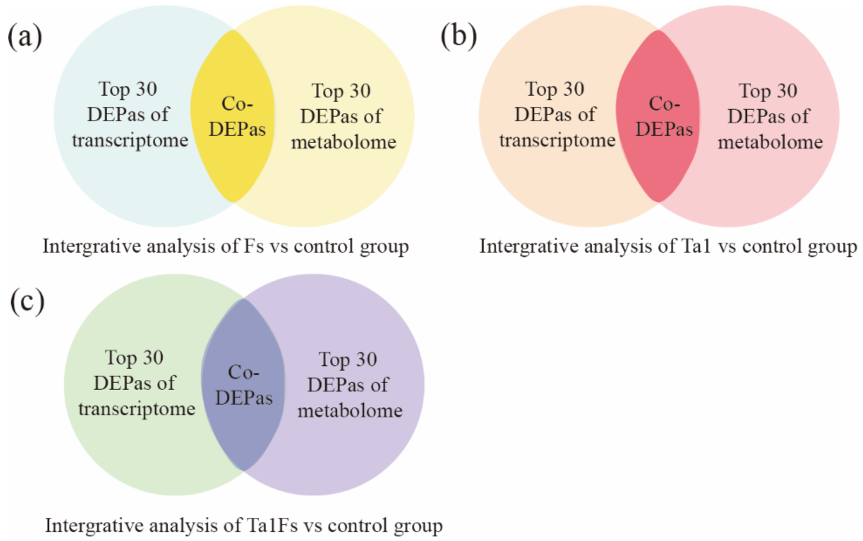

Figure S1 Schematic diagram of multi-omics analysis method. (a) Multi-omics analysis method of group Fs vs Control; (b) Multi-omics analysis method of group Ta1Fs vs Control; (c) Multi-omics analysis method of group Ta1 vs Control.

Sequence S1:

>ITS1/4 sequence of isolated *Trichoderma* spp.

ACTGACGCGGAGGGACATTACCGAGTTTACAACTCCCAAACCCAATGTGAACGTTACCAAA
CTGTTGCCTCGGCGGGGTCACGCCCCGGGTGCGTAAAAGCCCCGGAACCAGGCGCCCGCC
GGAGGAACCAACCAAACTCTTTCTGTAGTCCCCTCGCGGACGTATTTCTTACAGCTCTGAG
CAAAAATTCAAAATGAATCAAAACTTTCAACAACGGATCTCTTGGTTCTGGCATCGATGAA
GAACGCAGCGAAATGCGATAAGTAATGTGAATTGCAGAATTCAGTGAATCATCGAATCTTTG
AACGCACATTGCGCCCGCCAGTATTCTGGCGGGCATGCCTGTCCGAGCGTCATTTCAACCCT
GAACCCCTCCGGGGGATCGGCGTTGGGGATCGGGACCCCTCACCGGGTGCCGGCCCTGAA
ATACAGTGGCGGTCTCGCCGCAGCCTCTCCTGCGCAGTAGTTTGCACAACTCGCACCGGGA
GCGCGGCGCGTCCACGTCCGTAAAACACCCAACTTCTGAAATGTTGACCTCGGATCAGGTA
GGAATACCCGCTGAACTTAAGCATATCAAATTGGGGAGGAAA

Sequence S2:

>EF1-α sequence of isolated *Trichoderma* spp.

CACCCTGGGGAGGTCGAGATCCCAAGTACTATGTCACCGTCATTGGTATGTTTTGGACTCTT
CTCTCTAGACTATCGACATTCCAAGTCCGCCATTCTAACATGCTCTTCGCCACAGACGCTCC
CGGTCACCGTGATTTCATCAAGAACATAGATCACTGGTACCTCCCAGGCTGACTGCGCTATC
CTGATTATCGCTGCCGGTACTGGTGACGTTCGAGGCTGGTCTCCAAGGATGGC
